# Rapid and Long-Lasting Increase in Sites for Synapse Assembly during Late-Phase Potentiation in Rat Hippocampal Neurons

**DOI:** 10.1371/journal.pone.0007690

**Published:** 2009-11-06

**Authors:** Irina Antonova, Fang-Min Lu, Leonard Zablow, Hiroshi Udo, Robert D. Hawkins

**Affiliations:** 1 Department of Neuroscience, Columbia University, New York, New York, United States of America; 2 New York State Psychiatric Institute, New York, New York, United States of America; Vrije Universiteit Amsterdam, Netherlands

## Abstract

Long-term potentiation in hippocampal neurons has stages that correspond to the stages of learning and memory. Early-phase (10–30 min) potentiation is accompanied by rapid increases in clusters or puncta of presynaptic and postsynaptic proteins, which depend on actin polymerization but not on protein synthesis. We have now examined changes in pre- and postsynaptic puncta and structures during glutamate-induced late-phase (3 hr) potentiation in cultured hippocampal neurons. We find that (1) the potentiation is accompanied by long-lasting maintenance of the increases in puncta, which depends on protein synthesis, (2) most of the puncta and synaptic structures are very dynamic, continually assembling and disassembling at sites that are more stable than the puncta or structures themselves, (3) the increase in presynaptic puncta appears to be due to both rapid and more gradual increases in the number of sites where the puncta may form, and also to the stabilization of existing puncta, (4) under control conditions, puncta of postsynaptic proteins behave similarly to puncta of presynaptic proteins and share sites with them, and (5) the increase in presynaptic puncta is accompanied by a similar increase in presumably presynaptic structures, which may form at distinct as well as shared sites. The new sites could contribute to the transition between the early and late phase mechanisms of plasticity by serving as seeds for the formation and maintenance of new synapses, thus acting as local “tags” for protein synthesis-dependent synaptic growth during late-phase plasticity.

## Introduction

Various forms of synaptic plasticity including long-term potentiation (LTP) in the hippocampus have stages that correspond to the stages of learning and memory. Early-phase (<1 hr) synaptic plasticity is thought to involve the covalent modification of proteins in existing pre- or postsynaptic structures [1], [2]. By contrast, late-phase (>3 hr) plasticity is generally thought to involve a completely different type of mechanism, the protein synthesis-dependent remodeling of existing synapses and formation of new synapses [3], [4]. A good deal is now known about the mechanisms of *de novo* synapse formation during development, which involves a fairly extended program of pre- and postsynaptic changes coordinated by transynaptic signaling [5], [6]. However, less is known about the mechanisms of synapse formation during late-phase plasticity. A number of studies have found that synaptic components undergo continual assembly and disassembly, and late-phase plasticity somehow alters that balance [7]–[12]. However, it is not known whether the assembly and disassembly occur at random or fixed sites, and whether plasticity alters the number or dynamics of those sites.

In addition, relatively little is known about the transition from early- to late phase plasticity, how the late-phase mechanisms are first initiated, and how they are coordinated with the early-phase mechanisms. Some of the early changes are thought to act as local “tags”, allowing only sites that are active in the early phase to utilize newly synthesized gene products from the nucleus during the late phase [13], [14]. However, the putative tag has not yet been identified, and a variety of molecules have been proposed as candidates [15]–[17]. The tag might also consist of structural alterations, which begin soon after the induction of hippocampal LTP. Within minutes there are increases in spine size [18], [19], clusters or puncta of both postsynaptic glutamate receptors [20] and presynaptic vesicle-associated proteins, as well as sites where the pre- and postsynaptic puncta colocalize [21], [22]. Within tens of minutes there is outgrowth of new pre- and postsynaptic structures [23]–[26], and by 0.5 to 15 hrs new synapses are formed [26]–[29]. Moreover, the early presynaptic alterations appear to depend on retrograde signaling from the postsynaptic cells [26], [30], [31], as occurs during the early stages of *de novo* synapse formation. These results suggest that the structural alterations during early-phase potentiation may act as tags or initial steps in a program that can lead to stable synaptic growth during late-phase potentiation.

To begin to explore these ideas, we have examined changes in synaptic puncta and structures during long-lasting potentiation in hippocampal neurons. Early-phase potentiation is accompanied by rapid increases in puncta of presynaptic (synaptophysin and synapsin I) and postsynaptic (GluR1 and PSD-95) proteins, which are due to aggregation of protein from a more diffuse background and are dependent on NMDA receptor activation and actin polymerization but not on protein synthesis [20], [21]. To investigate the possible contribution of these events to late-phase potentiation, we have now addressed three questions: Do these early changes persist and become protein synthesis dependent? What is the nature of the changes? How might they contribute to the late phase? Our results suggest that the onset of potentiation is accompanied by rapid and long-lasting increases in the number of sites where synaptic puncta and structures are assembled. These new sites are much more stable than the puncta or structures themselves, and may contribute to the transition between the early and late phase mechanisms of plasticity by serving as seeds or tags for the protein synthesis-dependent assembly and maintenance of synaptic components during the late phase.

## Results

### Late-Phase Potentiation Is Accompanied by Long-Lasting Increases in Puncta of Presynaptic and Postsynaptic Proteins, Which Depend on Protein Synthesis

**Figure 1:**
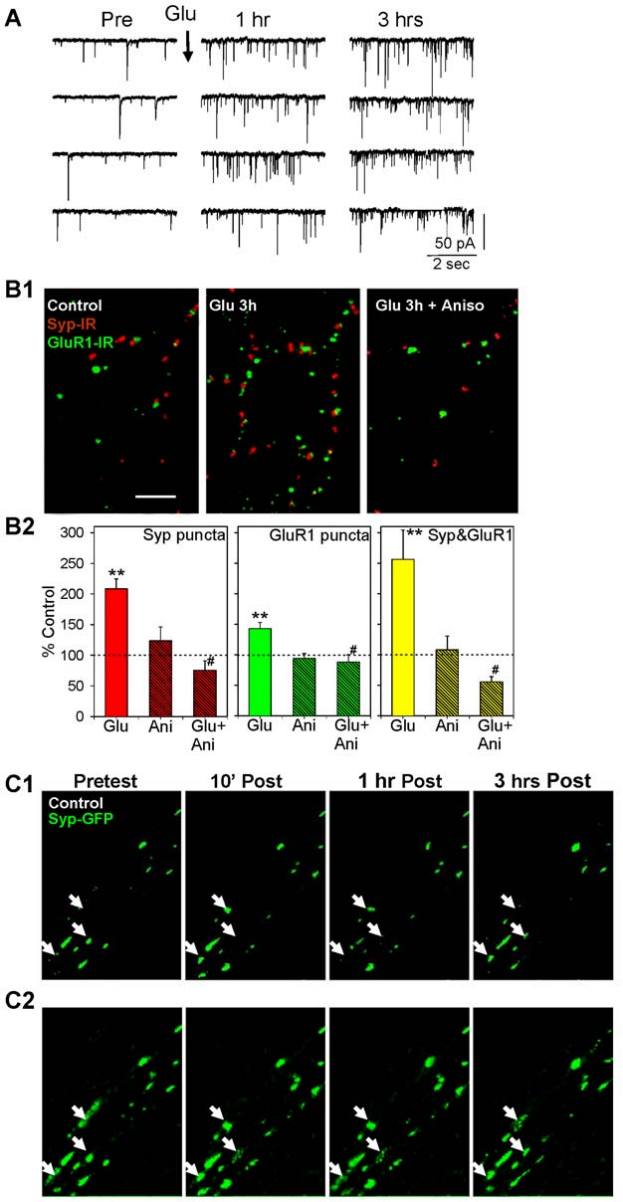
Glutamate-induced late-phase potentiation of the frequency of mEPSCs is accompanied by a protein synthesis-dependent increase in the number of clusters or puncta of synaptic proteins, which aggregate and disaggregate at fixed sites. A. Examples of spontaneous mEPSCs before (Pre) and 1 hr and 3 hrs after brief application of glutamate. B1. Examples of synaptophysin-immunoreactive (IR) puncta (red), GluR1-IR puncta (green), and colocalization (either yellow or adjacent red and green) in dishes fixed 3 hrs after brief application of saline (control), glutamate, or glutamate following pretreatment with anisomycin. Scale bar, 10 µM. B2. Average results from experiments like the ones shown in B1 (n = 9 dishes in each group). In this and subsequent figures the graphs show the means and SEMs, **  = p<0.01, *  = p<0.05, + = p<0.05 one-tail compared to control, #  = p<0.05, + = p<0.05 one-tail compared to no inhibitor. The average number of puncta in ten fields (94 µm×142 µm) per dish has been normalized to the number in comparable fields in control dishes from the same culture batch. The average control values per field were 35 (synaptophysin), 68 (GluR1), and 12 (colocalized). C1. Examples of synaptophysin-GFP fluorescent puncta before (Pretest) and 10 min, 1 hr, and 3 hrs after (Post) brief application of saline (control). C2. The same images as C1 (which were generated with an intensity threshold to facilitate recognition and counting of the puncta) without the threshold, showing that the puncta undergo continual aggregation, disaggregation, and reaggregation at fixed sites (the arrows indicate three examples).

Brief application of glutamate (200 µM in 0 Mg^2+^ saline for 1 min) produces rapid potentiation of evoked EPSCs and an increase in the frequency of spontaneous mEPSCs (miniature excitatory postsynaptic currents) in dissociated cultures of hippocampal neurons [19], [21], [32]. We found that the increase was maintained for more than 1 hr (X = 490% of pretest compared to 161% for saline controls, F[1,144] = 9.43, p<0.01 ), with no significant change in mEPSC amplitude. Furthermore, in experiments with stable recordings for 3 hrs, the increase in frequency was maintained at a fairly constant level for at least that long (X = 545%, t[2] = 3.44, p<0.05 one-tail vs. pretest) (Fig. 1A).

When the neurons were fixed 3 hrs after the glutamate application, there were also significant increases in the number of synaptophysin-immunoreactive (IR) puncta (t[16] = 6.31, p<0.01 compared to saline control), GluR1-IR puncta (t = 3.61, p<0.01), and sites where they colocalized (t = 3.37, p<0.01) (Fig. 1B). However, unlike the increases at 10 min [21], the increases at 3 hrs were all blocked by the protein synthesis inhibitor anisomycin (30 µM for 1 hr before and during the glutamate application) (p<0.05 for the glu x aniso interaction in each case). Anisomycin alone had no significant effect.

**Figure 2:**
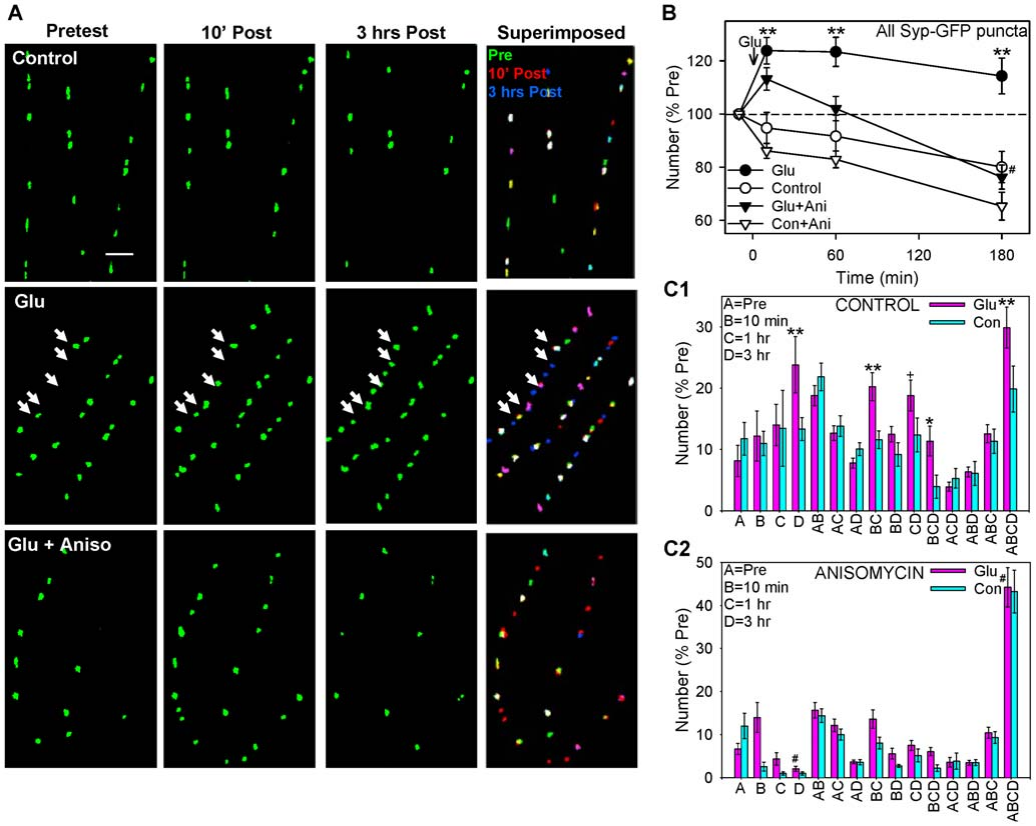
Glutamate produces a protein synthesis-dependent long-lasting increase in the number of synaptophysin-GFP puncta, which is due to a prolonged increase in the assembly of new puncta and also stabilization of existing puncta. A. Examples of synaptophysin-GFP fluorescent puncta before (Pretest), 10 min and 3 hrs after (Post) brief application of saline (control), glutamate, or glutamate following pretreatment with anisomycin. In the last column images from the 3 times in each experiment have been superimposed and color coded so that puncta that were present at different combinations of times are represented by different combinations of colors (pretest only green, 10 min only red, 3 hrs only blue, pretest and 10 min yellow, pretest and 3 hrs light blue, 10 min and 3 hrs purple, and all three times white). Scale bar, 10 µm. B. Average time course of changes in the total number of synaptophysin-GFP puncta (n = 17 control dishes, 30 glutamate, 12 anisomycin, 16 glutamate + anisomycin). There were significant overall effects of glutamate (F[1,71] = 23.40, p<0.01), anisomycin (F = 10.54, p<0.01), and the anisomycin x time interaction (F[2,142] = 3.96, p<0.05) in a 3-way ANOVA with one repeated measure (time). The number of puncta in one field (94 µm×142 µm) per dish has been normalized to the number before glutamate application (pretest) in each experiment. The average pretest value per field was 94, not significantly different between the groups by a 1-way ANOVA. C. Average number of puncta with different life histories: present only in the pretest (A), 10 min (B), 1 hr (C), 3 hrs (D), and various combinations of times (AB, AC, etc.) after glutamate or saline control, from the same experiments as B. There were significant overall effects of glutamate (F[1,71] = 8.55, p<0.01), anisomycin (F = 22.70, p<0.01), the glutamate x history interaction (F[14,994] = 1.66, p<0.05), and the anisomycin x history interaction (F = 8.17, p<0.01) in a 3-way ANOVA with one repeated measure (history). The symbols indicate the significance of planned comparisons of glutamate vs. control for each history in C1, and control vs. anisomycin (or the glutamate x anisomycin interaction) in C2.

These results suggest that late-phase potentiation is accompanied by a long-lasting increase in puncta of synaptic proteins, which depends on protein synthesis. However, these experiments could not address such questions as when and where the puncta assemble and/or disassemble, and whether plasticity alters the number or dynamics of those sites. To examine those questions we used recombinant adenovirus to express a synaptophysin-GFP (Green Fluorescent Protein) fusion protein, which permits imaging of individual puncta over time in living neurons. Replicating the synaptophysin-IR results, we found that brief application of glutamate produced a rapid increase in the number of synaptophysin-GFP puncta that was maintained for at least 3 hrs (F[1,213] = 19.07, p<0.01 compared to saline control), and anisomycin blocked the increase at 3 hrs (F = 3.43, p<0.05 one-tail for the glu x aniso interaction) but not at 10 min (Fig. 2A,B). Anisomycin alone had no significant effect compared to test-alone control, which exhibited a decrease that may be due to nonspecific effects the testing procedure.

### The Increase in Puncta of Presynaptic Proteins Appears to Be Due to Rapid and More Gradual Increases in the Number of Sites where the Puncta May Form, as well as Stabilization of Existing Puncta

We then followed the “life histories” of individual puncta of synaptophysin-GFP over 3 hrs, and examined the effects of both glutamate and anisomycin (Figs. 1C, 2A,C). Under saline control conditions, individual puncta were present at all possible combinations of pretest (A), 10 min posttest (B), 1 hr posttest (C), and 3 hr posttest (D), including ones that involved disassembling and reassembling at the same site (e.g. AC, ABD, BD, etc.). The images in these figures were generated with an intensity threshold to facilitate recognition and quantification of the puncta. When we examined changes in the subthreshold surround, we found that the puncta aggregate, disaggregate, and reaggregate at the same sites (Fig. 1C2). Collectively, these results suggest that the assembly and disassembly of puncta is due to continual aggregation and disaggregation at fixed sites that are more stable than the puncta themselves.

Brief application of glutamate produced significant increases in the number of puncta that assembled for the first time 10 min (BC and BCD) as well as 1 hr (CD) and 3 hrs (D) after the glutamate, and also puncta that were stable for the entire experiment (ABCD) (p<0.05 compared to saline control in each case) (Fig. 2C). Although anisomycin alone had no significant effect on changes in the total number of puncta, it had an effect on their life histories, increasing the number of stable puncta (ABCD) and decreasing all other types. In addition, anisomycin blocked the glutamate-induced increase in puncta that assembled for the first time at 3 hrs (D) and also blocked or occluded the increase in stable puncta (ABCD) (p<0.05 one-tail for the glu x aniso interaction in each case). These results suggest that the long-lasting increase in synaptophysin-GFP puncta is due to a prolonged increase in the assembly of new puncta and also stabilization of existing puncta, both of which involve protein synthesis.

**Figure 3:**
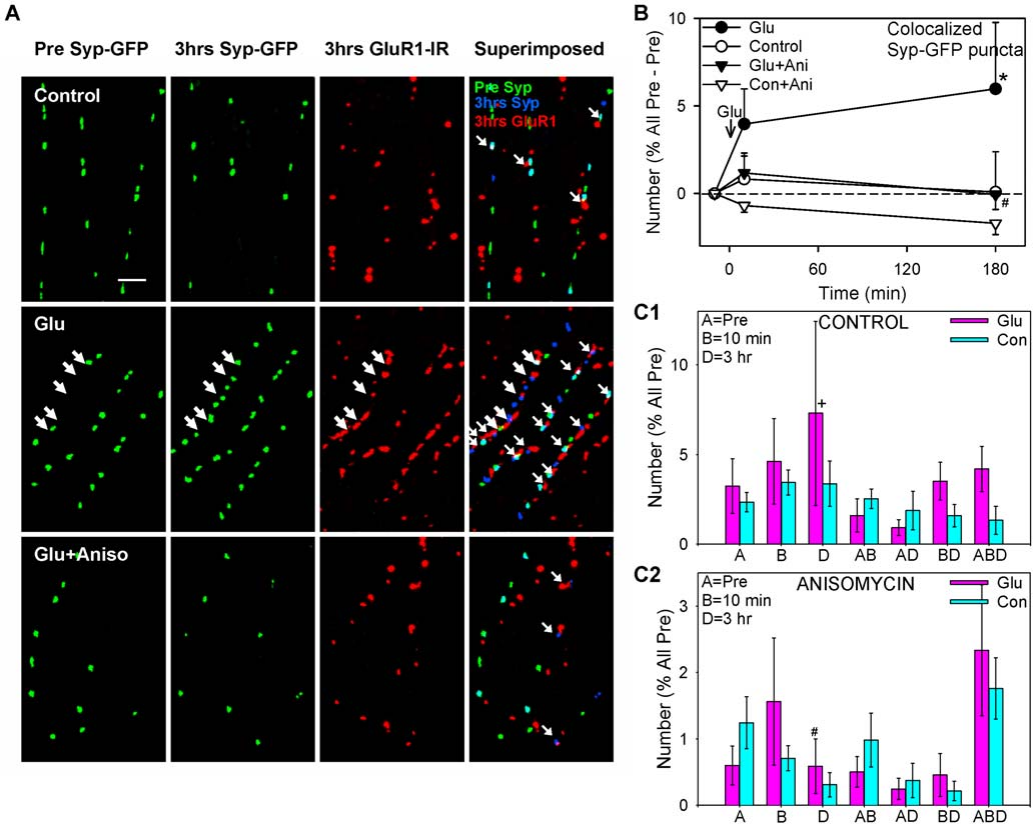
Some of the synaptophysin-GFP puncta colocalize with GluR1-IR puncta at 3 hrs, and those puncta behave similarly to all Syp-GFP puncta. A. Examples from the same experiments as Fig. 2A showing synaptophysin-GFP fluorescent puncta (green) before and 3 hrs after, and GluR1-IR puncta (red) 3 hrs after brief application of saline (control), glutamate, or glutamate following pretreatment with anisomycin. In the last column the three images in each experiment have been superimposed and color coded to show colocalization of Syp-GFP puncta from the pretest (green) and 3 hrs (blue) with GluR1-IR puncta at 3 hrs (red). Thus Syp-GFP puncta from the pretest that colocalized with GluR1-IR puncta are either yellow or adjacent green and red, those from 3 hrs are either purple or adjacent blue and red, and those from both times are either white or adjacent light blue and red (small arrows). B. Average time course of changes in the number of Syp-GFP puncta at any time that colocalized with GluR1-IR puncta at 3 hrs (n = 8 control dishes, 10 glutamate, 10 anisomycin, 7 glutamate + anisomycin). Because there were sometimes no colocalized Syp-GFP puncta in the pretest, the number of colocalized Syp-GFP puncta has been normalized as the percentage of all Syp-GFP puncta in the pretest minus the colocalized pretest value in each experiment, so that zero represents no change. The average colocalized pretest value was 6.7, not significantly different between the groups by a 1-way ANOVA. C. Average number of colocalized Syp-GFP puncta that were present only in the pretest (A), 10 min (B), 3 hrs (D), and various combinations of times (AB, AD, etc.) after glutamate or saline control, from the same experiments as B.

To determine which of the synaptophysin-GFP puncta (e.g. new vs. old, stable vs. not) might be at synaptic sites, we repeated live imaging of synaptophysin-GFP and performed retrospective immunocytochemistry for GluR1 after 3 hrs (Fig. 3A). Like synaptophysin-IR puncta (Fig. 1B), only some of the synaptophysin-GFP puncta colocalized with GluR1-IR puncta. When we restricted our analysis to synaptophysin-GFP puncta at any time that colocalized with the GluR1-IR puncta at 3 hrs and therefore might be synaptic (Fig. 3B,C), the results were qualitatively similar to those described above for all synaptophysin-GFP puncta (the 1 hr posttest was omitted to limit the number of combinations in this analysis). In particular, the percentages of synaptophysin-GFP puncta that were new or old, stable or not were about the same for the colocalized puncta as for all puncta. However, there was a smaller decrease in the test-alone control group (Fig. 3B), suggesting that most of the decrease in Fig. 2B was due to loss of nonsynaptic puncta. There was also a relatively greater increase (or smaller decrease) in the total number of colocalized synaptophysin-GFP puncta between 10 min and 3 hrs, and a greater increase in the number of colocalized puncta that assembled for the first time at 3 hrs (Fig. 3C). As for all synaptophysin-GFP puncta, both of those increases were blocked by anisomycin (p<0.05 in each case).

**Figure 4:**
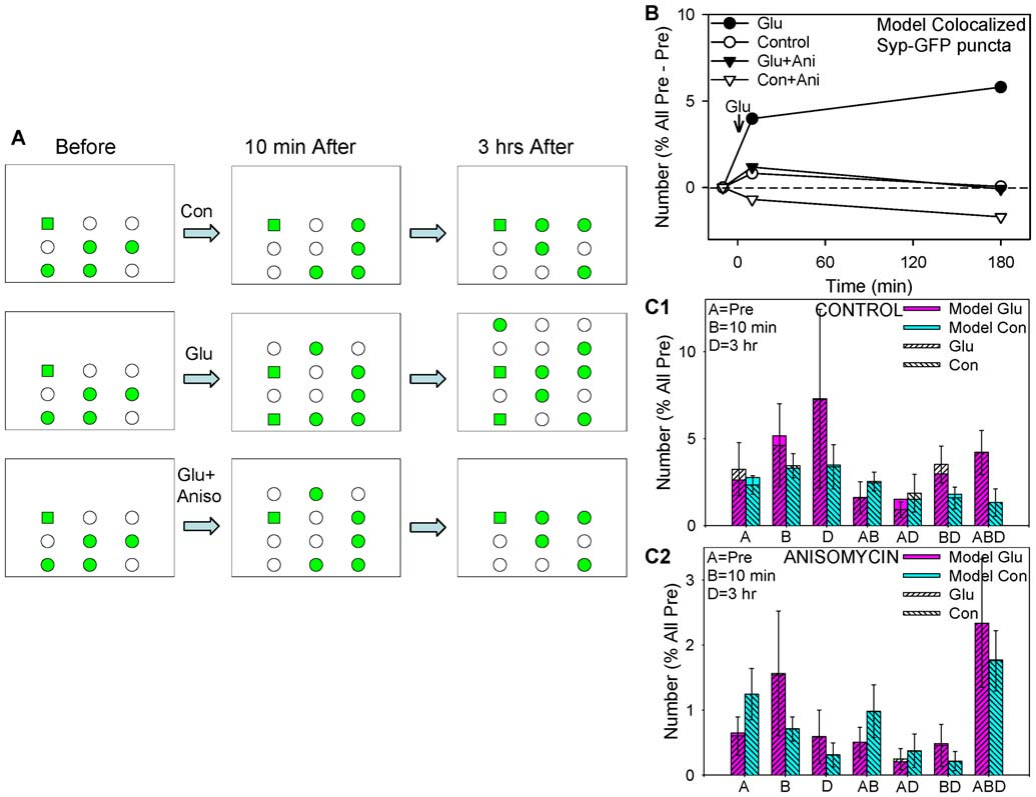
A quantitative model of the data on colocalized synaptophysin-GFP puncta. A. Cartoon of the model. There are a fixed number of sites, some of which are filled (green) and some of which are empty (white). Under control conditions, most sites fill and empty independently with constant probabilities. Glutamate produces rapid and more gradual increases in the total number of sites, and makes some previously filled sites stable (squares). Anisomycin interferes with the glutamate-induced stabilization, gradual increase in sites, and maintenance of the rapid increase. B,C. Quantitative simulations based on this model fit the actual data for the colocalized Syp-GFP puncta very well.

These results suggest the model illustrated qualitatively in Fig. 4A and described more quantitatively in Table 1. There are a fixed number of sites where synaptophysin-GFP puncta may form, some of which are filled (green) and some of which are empty (white). Under control conditions, most sites fill and empty independently with constant probabilities. To begin to test whether this simple model was plausible we first performed a quantitative simulation of the control data, and found that the model could fit both the total number and life histories of the puncta quite well (Fig. 4B,C).

**Table 1:** Colocalized synaptophysin-GFP puncta.

	Control	Glutamate	Anisomycin	Glu+Aniso
N1	186	281	53	122
N2	0	33	34	59
P1	.47	.38	.44	.44
P2	.34	.29	.40	.45
P3	.994	1.008	.965	.964

Parameters of the model used to fit the data on colocalized synaptophysin-GFP puncta. The model starts with 100 occupied sites, of which N2 are stable for the entire experiment, and N1 unoccupied sites. In each 10 min time interval, the probability of a site going from unoccupied to occupied is P2, the probability of going from occupied to unoccupied is (1 - P1), and the probability of going from present to absent (starting at 10 min) is (1 - P3). An increase in N1 (for example, comparing glutamate to control) corresponds to a rapid increase in sites at 10 min, an increase in P3 corresponds to a gradual increase (or a smaller decrease) from 10 min to 3 hrs, and an increase in N2 corresponds to an increase in stable sites.

We next asked whether changes in the parameters of the model could account for the glutamate and anisomycin data. In the model, glutamate and anisomycin might affect either the probabilities of sites filling and emptying or the number of sites, compared to control. However, we could not fit the glutamate data by changing the probabilities of sites filling and emptying. Instead, the fit was best if glutamate was assumed to produce an increase in the total number of sites, with two phases: a rapid increase between 0 and 10 min, and a more gradual increase between 10 min and 3 hrs. In addition, glutamate converted some sites that were filled in the pretest into stable sites (squares). Likewise, the fit to the anisomycin data was best if anisomycin was assumed to block the gradual increase in the number of sites between 10 min and 3 hrs, and also to interfere with maintenance of the rapid increase in sites as well as the increase in stable sites. When the probabilities of sites filling and emptying and the number of sites in the model were both allowed to vary, changes in the probabilities made a relatively modest contribution to the best fit for the glutamate and anisomycin results, whereas changes in the number of sites made a much larger contribution (Table 1). Quantitative simulations based on this model fit the data for the colocalized synaptophysin-GFP puncta very well (Fig. 4B,C), and the fit for all puncta was also good (not shown).

### Under Control Conditions, Puncta of Postsynaptic Proteins Behave Similarly to Puncta of Presynaptic Proteins and Share Sites with Them

These results suggest that the long-lasting increase in synaptophysin puncta involves the formation and maintenance of new presynaptic sites that are more stable than the puncta themselves. However, they provide little information about the nature of those sites. To begin to investigate that question we first examined whether there are similar postsynapric sites, and if so how they relate to the presynaptic sites. In our initial experiments, we used recombinant adenovirus to express a PSD95-RFP (Red Fluorescent Protein) fusion protein, and examined the distribution of the protein under control conditions.

**Figure 5:**
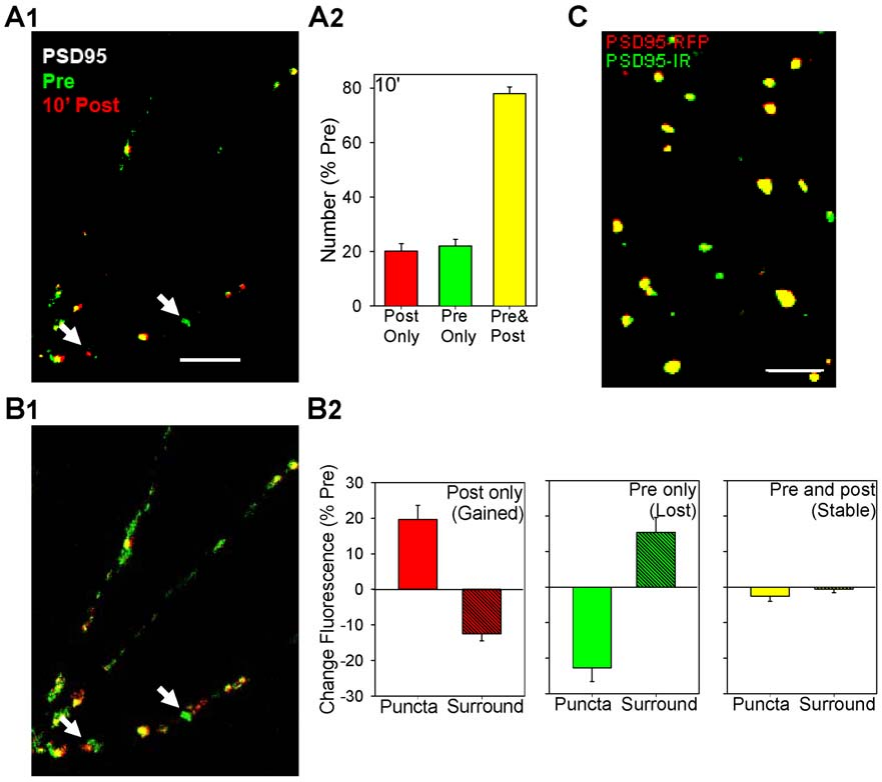
Puncta of PSD95-RFP aggregate and disaggregate like puncta of synaptophysin-GFP under control conditions. A1. Example of PSD95-RFP puncta before (Pre, green) superimposed on those 10 min after (red) brief application of saline (control). Puncta that were present in the posttest only appear red, those that were present in the pretest only appear green, and those that were present at both times appear yellow. The arrows indicate two examples. Scale bar, 10 µm. A2. Average number of puncta that were present in the posttest only (red), the pretest only (green), or both times (yellow). The number of puncta in the field have been normalized to the number on the pretest in each experiment (average pretest value  = 73, n = 21). B1. The same image as A1 without the threshold used to discriminate puncta from more diffuse background fluorescence. B2. Average changes in fluorescence of the puncta and the surrounding area within 5 µm for puncta that were present in the posttest only, the pretest only, or both times. The changes in fluorescence have been normalized to the total fluorescence of the puncta and surround on the pretest in each experiment. C. Example of puncta of PSD95-RFP (red), PSD95-IR (green), and colocalization (yellow). Scale bar, 10 µm. Most puncta colocalized but a few of each type did not, suggesting that the PSD95 monoclonal antibody did not simply recognize PSD95-RFP.

**Figure 6:**
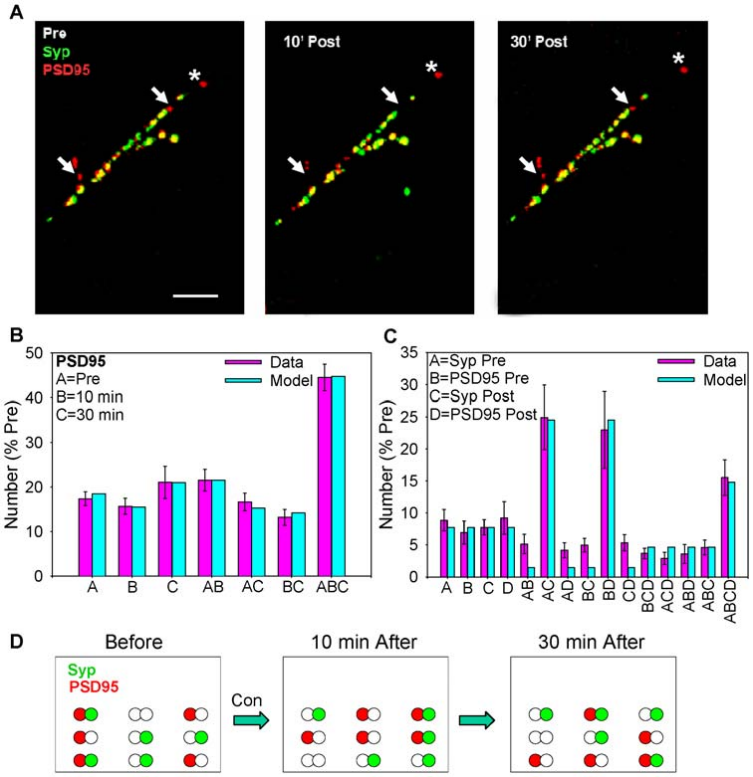
Puncta of PSD95-RFP and synaptophysin-GFP assemble and disassemble with similar parameters but independently at shared sites. A. Examples of synaptophysin-GFP (green), PSD95-RFP (red), and colocalization (either yellow or adjacent green and red) before (Pre), 10 min, and 30 min after brief application of saline (control). The arrows indicate examples of PSD95-RFP puncta that were present in the pretest, disassembled at 10 min, and reassembled at 30 min, and the asterisk indicates one that moved. Scale bar, 10 µm. B. Average number of PSD95-RFP puncta that were present only in the pretest (A), 10 min (B), 30 min (C), and various combinations (AB, AC, etc.), compared to results of a model like the one for synaptophysin-GFP shown in Fig. 4A. The number of puncta in the field has been normalized to the number on the pretest in each experiment (average pretest value  = 114, n = 24). C. Average number of sites with only synaptophysin in the pretest (A), PSD95 in the pretest (B), synaptophysin in the posttest (C), PSD95 in the posttest (D), and various combinations (AB, AC, etc.), compared to results of the model illustrated in D. Data for the 10 min and 30 min posttests were similar, and have been pooled. The number of sites of each type in the field has been normalized to the sum of synaptophysin-GFP and PSD95-RFP puncta on the pretest in each experiment (average pretest values  = 48 synaptophysin and 51 PSD95, n = 11). D. Cartoon of the model used to fit the data in C. Puncta of synaptophysin and PSD95 assemble and disassemble independently with the same parameters at shared sites, which are more stable than the puncta themselves.

Time-lapse imaging revealed that puncta of PSD95-RFP continually assembled (red, 10′ post only) and disassembled (green, pre only) (Fig. 5A). Examination of changes in fluorescence in the subthreshold surround showed that for the puncta that assembled (post only) there was a decrease in fluorescence in the surrounding area (t[17] = 6.12, p<0.01), consistent with aggregation into the puncta (Fig. 5B). Conversely, for puncta that disassembled (pre only) there was an increase in fluorescence in the surrounding area (t[18] = 3.63, p<0.01), consistent with disaggregation. When we followed individual puncta over time they were present at all possible combinations of pretest, 10 min posttest, and 30 min posttest, including ones that involved disassembling and reassembling at the same site (Fig. 6A,B). These results suggest that puncta of PSD95-RFP continually aggregate and disaggregate at fixed sites. Furthermore, their “life histories” could be fit very well by the model described above for synaptophysin-GFP puncta (Fig. 6B) with similar time constants for assembly and disassembly of the puncta (P1 = .55 and P2 = .39; see Table 1). In all of these respects, the behavior of the PSD95-RFP puncta was similar to that of synaptophysin-GFP puncta. PSD95-RFP puncta colocalized very well with PSD95-IR puncta (Fig. 5C), suggesting that endogenous PSD95 may also behave similarly.

When we coexpressed synaptophysin-GFP and PSD95-RFP puncta of the two proteins were sometimes colocalized but assembled and disassembled separately, with all possible combinations of assembly and disassembly of the two types of puncta occurring at individual sites (Fig. 6A,C). These data were fit fairly well by a model in which puncta of the pre- and postsynaptic proteins assemble and disassemble independently at shared (presumably synaptic) sites, which are more stable than puncta of either type (Fig. 6C,D). Furthermore, although the colocalization of pre- and postsynaptic puncta is fairly low at any given time, the colocalization of pre- and postsynaptic sites is much higher (100% in this simple model).

### The Changes in Puncta Are Accompanied by Similar Changes in Presumably Presynaptic Structures

**Figure 7:**
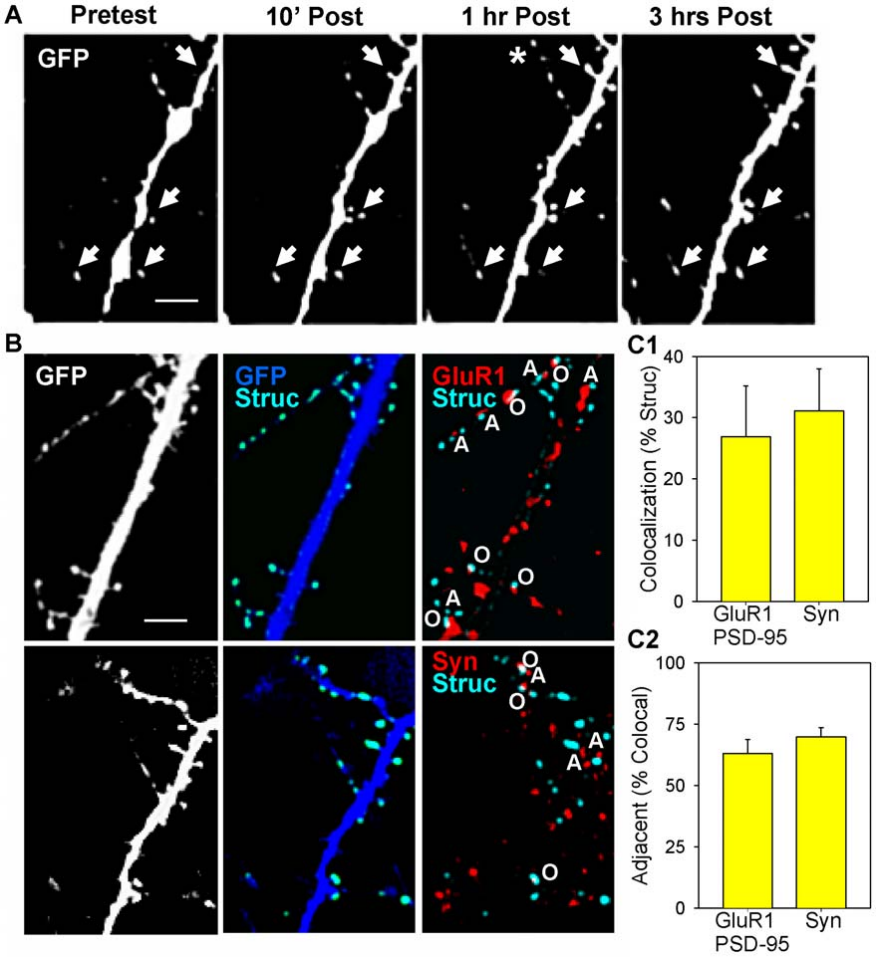
Examples of changes in small structures, automatic identification, and colocalization with puncta of synaptic proteins. A. Neurons transfected with GFP, which labels the entire cell, before (Pretest) 10 min, 1 hr, and 3 hrs after brief application of saline (control). The arrows indicate examples of changes in small structures, and the asterisk indicates outgrowth of a filopodium. Scale bar, 10 µm. B. Automatic identification of small structures, and colocalization with synaptic proteins. Left. Original images of neurons transfected with GFP (white). Scale bar, 10 µm. Middle. Automatic identification of small structures (Struc, light blue) by subtracting a smoothed image from the original image (dark blue). Right. Some of the small structures colocalize with puncta that are immunoreactive (IR) for GluR1 (red, top) or synapsin (red, bottom). Colocalized structures are either overlapping (O) white or adjacent (A) light blue and red. C1. Average percentage of small structures that colocalized with puncta of the postsynaptic proteins GluR1 (n = 3 dishes) or PSD95 (n = 4), or the presynaptic protein synapsin (n = 6). Results for GluR1 and PSD95 were similar, and have been pooled. The number of colocalized structures in one field (94 µm×142 µm) per dish has been normalized to the total number of structures in that field (average value = 97). C2. Average percentage of colocalized small structures that were adjacent (within 1 µm) but did not overlap with puncta of GluR1, PSD95, or synapsin.

We next examined whether the sites might correspond to morphological structures such as presynaptic varicosities and postsynaptic spines, and whether those structures behave similarly to the puncta or are more stable. To address those questions we used recombinant adenovirus to express GFP, which labels the entire neuron, although the axons were usually too thin to be visible (Fig. 7). We observed a variety of morphological changes including extension and retraction of processes (asterisk in Fig. 7A). However, by far the most common changes were assembly and disassembly of small structures that might be spines on dendrites or varicosities on axons (arrows). Because those structures are also the most likely sites for puncta of synaptic proteins, we decided to focus our analysis on them.

To identify the small structures objectively, we developed an automatic algorithm that subtracted a smoothed image from the original image (light blue in Fig. 7B). Approximately 30 percent of the structures colocalized with puncta that were immunoreactive for the synaptic proteins GluR1, synapsin I (red in Fig. 7B), and PSD-95, supporting the idea that the structures may be synaptic (Fig. 7C1). That percentage is comparable to colocalization of puncta of pre- and postsynaptic proteins in similar studies ([31] and Figs. 1, 3, 6).

The colocalized structures might be either postsynaptic spines or presynaptic varicosities. Those alternatives were often difficult to distinguish in the GFP images because the axons were not visible, either because they were too thin or lay on a dendrite. In that case, what appeared to be the bulbous head of a spine might actually have been a presynaptic varicosity or vice versa. To attempt to distinguish between those possibilities, we divided the structures into ones that overlapped (at least in part) and ones that were adjacent to puncta of GluR1-IR, PSD95-IR, or synapsin-IR. In three dimensions, structures that overlap might be either pre- or postsynaptic, but structures that are adjacent to puncta of the postsynaptic proteins GluR1 or PSD-95 are presumably presynaptic, and structures that are adjacent to puncta of the presynaptic protein synapsin are presumably postsynaptic [31], [33]. Roughly half of the structures that colocalized with puncta of GluR1-IR or PSD95-IR were adjacent to them, and a similar percentage of the structures that colocalized with synapsin-IR were adjacent (Fig. 7C2). These results suggest that the structures include both presynaptic varicosities and postsynaptic spines, in about equal numbers.

**Figure 8:**
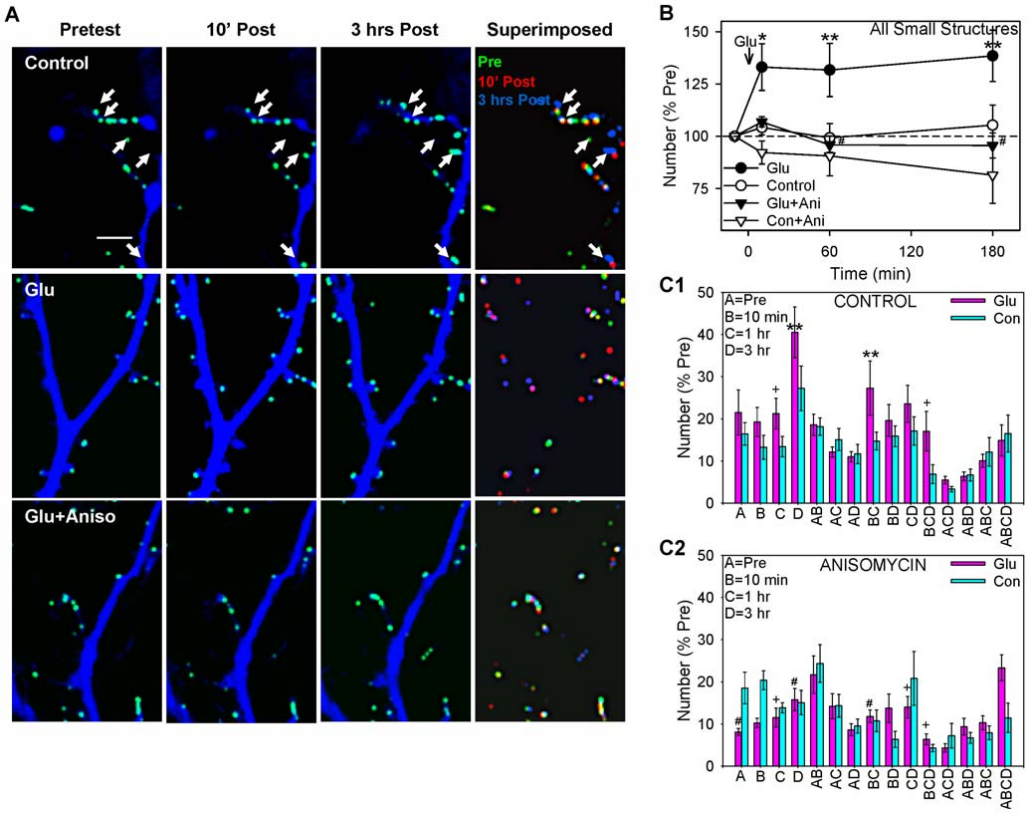
Glutamate produces a protein synthesis-dependent long-lasting increase in the number of small structures, which is due to a prolonged increase in the assembly of new structures. A. Examples of small structures (light blue) before (Pretest), 10 min and 3 hrs after (Post) brief application of saline (control), glutamate, or glutamate following pretreatment with anisomycin. In the last column images from the 3 times in each experiment have been superimposed and color coded so that structures that were present at different combinations of times are represented by different combinations of colors (pretest only green, 10 min only red, 3 hrs only blue, pretest and 10 min yellow, pretest and 3 hrs light blue, 10 min and 3 hrs purple, and all three times white). Scale bar, 10 µm. B. Average time course of changes in the total number of small structures (n = 14 control dishes, 14 glutamate, 6 anisomycin, 6 glutamate + anisomycin). There were significant overall effects of glutamate (F[1,36] = 4.12, p<0.05) and anisomycin (F = 5.55, p<0.05) in a 3-way ANOVA with one repeated measure (time). The number of structures in one field (94×142 µm) per dish has been normalized to the number before glutamate application (pretest) in each experiment. The average pretest value per field was 119, not significantly different between the groups by a 1-way ANOVA. C. Average number of structures with different life histories: present only in the pretest (A), 10 min (B), 1 hr (C), 3 hrs (D), and various combinations of times (AB, AC, etc.) after glutamate or saline control, from the same experiments as B. There were significant overall effects of anisomycin (F[1,36] = 8.69, p<0.01), the glutamate x anisomycin interaction (F = 3.87, p<0.05), and the anisomycin x history interaction (F[14,504] = 2.26, p<0.01) in a 3-way ANOVA with one repeated measure (history).

Under control conditions, there was little change in the number of structures over the course of 3 hrs (Fig. 8A,B). Brief application of glutamate produced an increase in the number of small structures within 10 min (F[1,108] = 5.30, p<0.05 compared to saline control), which was then maintained for 3 hrs (F = 7.01, p<0.01). Anisomycin significantly reduced the increase at 3 hrs (F = 7.06, p<0.01). Anisomycin alone had no significant effect compared to test-alone control.

Like puncta of synaptophysin-GFP, under control conditions individual small structures were present in all combinations of the pretest (A), 10 min posttest (B), 1 hr posttest (C), and 3 hr posttest (D), including ones that involved disassembling and reassembling at the same site (e.g. AC, ABD, BD, etc.) (Fig. 8A,C). These results suggest that the structures continually assemble and disassemble at fixed sites that are more stable than the structures themselves. Brief application of glutamate produced increases in the number of structures that assembled for the first time 10 min (BC and BCD), 1 hr (C), and 3 hrs (D) after the glutamate (p<0.05 one-tail compared to saline control in each case). Anisomycin blocked all of these glutamate-induced increases in structures (p<0.05 one-tail in each case).

**Figure 9:**
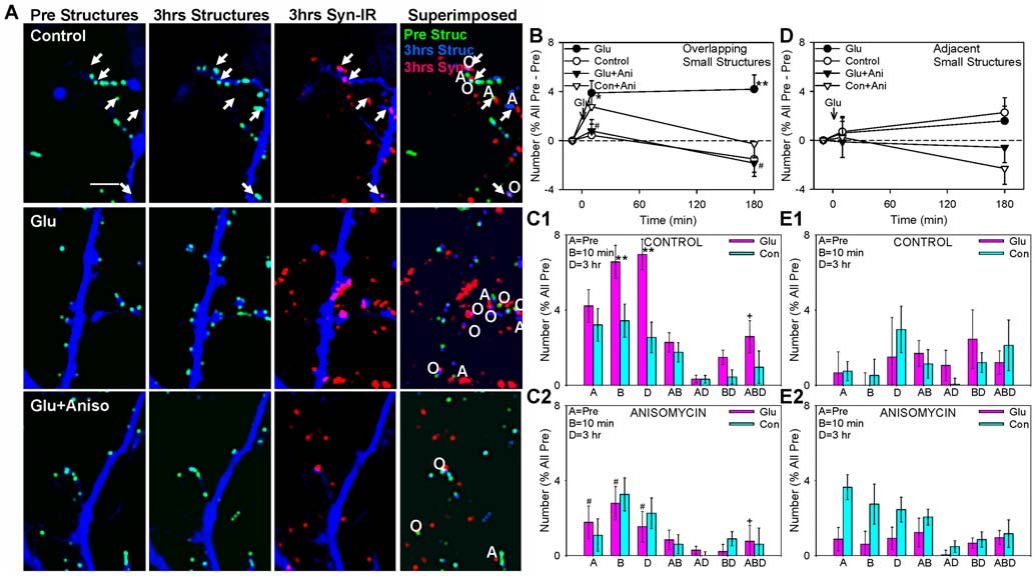
Some of the small structures overlap with synapsin-IR puncta at 3 hrs, and those structures behave generally similarly to all structures. A. Examples of small structures (light blue) before and 3 hrs after, and synapsin-IR puncta (red) 3 hrs after brief application of saline (control), glutamate, or glutamate following pretreatment with anisomycin. In the last column the three images in each experiment have been superimposed to show colocalization of structures from the pretest (green) and 3 hrs (blue) with synapsin-IR at 3 hrs (red). Thus structures from the pretest that colocalized with synapsin-IR puncta are either overlapping (O) yellow or adjacent (A) green and red, those from 3 hrs are either overlapping purple or adjacent blue and red, and those from both times are either overlapping white or adjacent light blue and red. B. Average time course of changes in the number of small structures at any time that overlapped with synapsin-IR puncta at 3 hrs (n = 6 control dishes, 6 glutamate, 6 anisomycin, 6 glutamate + anisomycin). There was a significant glutamate x anisomycin interaction (F[1,19] = 12.76, p<0.01) and an anisomycin x time interaction (F = 3.93, p<0.05 one-tail) in a 3-way ANCOVA with one repeated measure (time) and the pretest as covariate. The number of overlapping structures has been normalized as the percentage of all structures in the pretest minus the overlapping pretest value in each experiment. The average overlapping pretest value was 5.4, not significantly different between the groups by a 1-way ANOVA. C. Average number of overlapping structures that were present only in the pretest (A), 10 min (B), 3 hrs (D), and various combinations of times (AB, AD, etc.) after glutamate or saline control, from the same experiments as B. There were significant overall effects of glutamate (F[1,20] = 3.02, p<0.05 one-tail), anisomycin (F = 9.69, p<0.01), the glutamate x anisomycin interaction (F = 3.59, p<0.05 one-tail), the anisomycin x history interaction (F[6,120] = 3.04, p<0.01), and the 3-way interaction (F = 3.04, p<0.01) in a 3-way ANOVA with one repeated measure (history). D,E. Time course and life histories of small structures that were adjacent to synapsin-IR puncta, from the same experiments as B and C. There were no significant effects of glutamate or anisomycin in planned comparisons of the different experimental treatments. The average pretest values for the overlapping and adjacent structures were not significantly different.

To examine the relationship between the small structures and puncta of synaptic proteins more directly, we repeated live imaging of the structures and performed retrospective immunocytochemistry for the presynaptic protein synapsin-I after 3 hrs (Fig. 9A). When we restricted our analysis to small structures at any time that colocalized with the synapsin-IR puncta at 3 hrs and therefore might be at synaptic sites, the results were qualitatively similar to those described above for all structures (not shown). The small structures probably include both postsynaptic spines and presynaptic varicosities (Fig. 7), which might behave differently. To attempt to address that question, we divided the colocalized structures into ones that overlapped (at least in part) and ones that were adjacent to synapsin-IR puncta. Under control conditions, both overlapping and adjacent structures continually assembled and disassembled. However, almost all of the changes in structures during potentiation were attributable to changes in the overlapping (presumably mostly presynaptic) structures. There was a gradual protein synthesis-dependent increase in adjacent (presumably postsynaptic) structures, but no difference between the glutamate and control groups (Fig. 9B,D). These results suggest that changes in the small structures during potentiation were mostly due to changes in presynaptic varicosities.

The overlapping (“presynaptic”) structures behaved similarly to all structures (Figs. 8, 9) in most ways. Brief application of glutamate produced an increase in the number of overlapping structures within 10 min (F[1,38] = 6.06, p<0.05 compared to saline control), which was then maintained for 3 hrs (F = 16.43, p<0.01). Anisomycin significantly reduced the increase at 10 min (F = 4.89, p<0.05) as well as 3 hrs (F = 18.39, p<0.01). Anisomycin alone had no significant effect compared to test-alone control. Similarly, when we examined the “life histories” of the overlapping structures, we found that brief application of glutamate produced significant increases in the number of structures that assembled for the first time 10 min (B) and 3 hrs (D) after the glutamate, and it also increased the number of stable structures (ABD) (p<0.05 one-tail compared to saline control in each case). Anisomycin blocked all of these glutamate-induced changes (p<0.05 one-tail in each case).

In some ways the overlapping structures behaved more like the synaptophysin-GFP puncta than like all structures, suggesting that presynaptic structures and puncta may assemble and disassemble together as a unit. Thus, glutamate increased the stability of overlapping structures and synaptophysin puncta, but not all structures. However, the overlapping structures and synaptophysin puncta behaved differently in other ways. For example, anisomycin blocked the glutamate-induced increase in overlapping structures but not synaptophysin puncta at 10 min as well as 3 hrs. In addition, anisomycin alone increased the number of stable synaptophysin puncta but not structures. Furthermore, although the data on synaptophysin puncta and overlapping structures could be fit quite well by the same general model, the control parameters were different and the sites underwent different plasticity in the models for the two sets of data (Table 2). These results suggest that the rules for the synaptophysin puncta and overlapping structures are similar but not identical and they may assemble and disassemble independently of each other, perhaps at distinct as well as shared sites.

**Table 2:** Overlapping small structures.

	Control	Glutamate	Anisomycin	Glu+Aniso
N1	449	564	609	546
N2	12	23	17	19
P1	.35	.37	.40	.31
P2	.14	.15	.30	.15
P3	.970	.987	.974	.961

Parameters of the model used to fit the data on overlapping small structures. See Table 1 legend.

**Figure 10:**
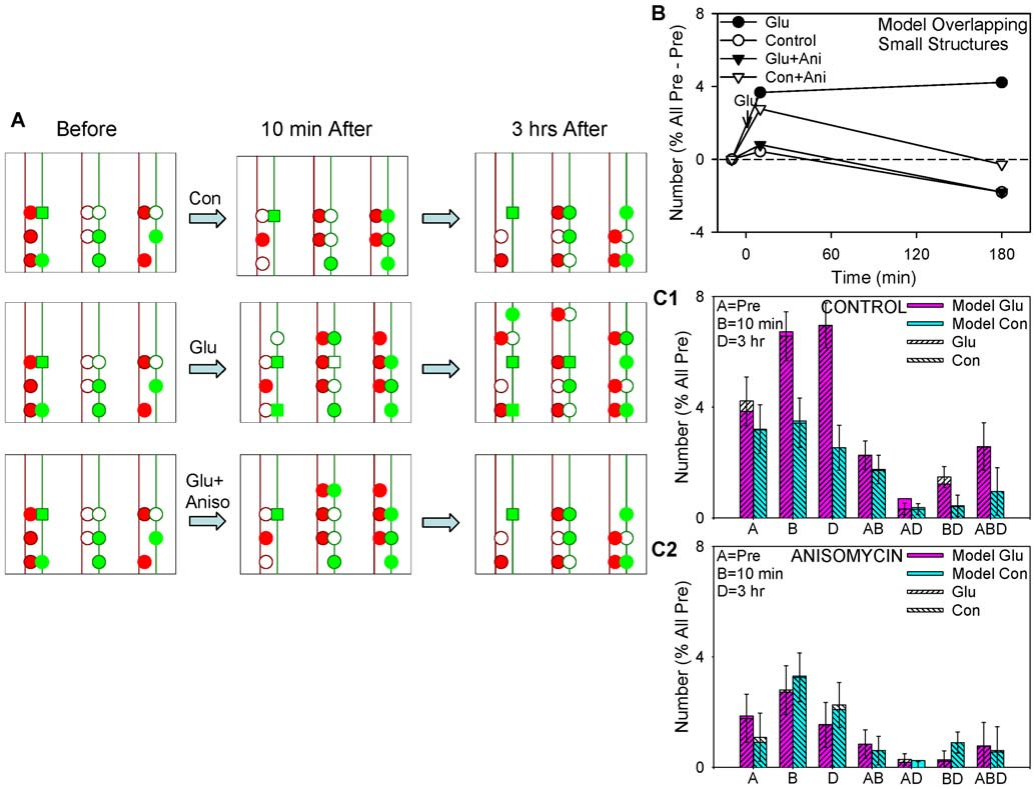
A quantitative model of the data on overlapping structures. A. Cartoon of a more general model for the data on puncta of the presynaptic proteins synaptophysin-GFP and synapsin-IR (green), the postsynaptic proteins PSD95-RFP and GluR1-IR (red), and pre- and postsynaptic small structures (green or red rims). See the text for details. B,C. Quantitative simulations based on this model fit the actual data for the overlapping (presynaptic) structures very well.

Together with the results on puncta of synaptophysin (Fig. 4) and PSD-95 (Fig. 6), these results suggest the model illustrated in Fig. 10A. There are a fixed number of sites where puncta of both presynaptic (green) and postsynaptic (red) proteins may form, some of which are filled (colored) and some of which are empty (white). Pre- and postsynaptic small structures (colored rims) may form at some of the same sites and perhaps also at additional sites. Under control conditions, most puncta and structures assemble and disassemble independently with constant probabilities, and a small number are stable (squares). Like the presynaptic puncta, glutamate produces an increase in the total number of sites where presynaptic structures may form, with two phases: a rapid increase between 0 and 10 min, and a more gradual increase between 10 min and 3 hrs. In addition, glutamate converts some sites that were occupied in the pretest into stable sites. Anisomycin reduces all of these actions of glutamate, but has less of an effect on the rapid increase in presynaptic puncta. The data for the postsynaptic puncta are less complete, but they seem to behave similarly to the presynaptic puncta: pre- and postsynaptic puncta assemble and disassemble at shared sites under control conditions (Fig. 6C,D), and they show similar increases 10 min and 3 hrs after potentiation ([21] and Fig. 1B). By contrast, the adjacent (“postsynaptic”) structures do not change significantly during potentiation.

Quantitative simulations based on these ideas fit the actual data for the overlapping (“presynaptic”) structures very well (Fig. 10B,C), and the fit for all structures is also good (not shown). The quantitative model we used for the overlapping structures is the same as the one we used for the puncta of synaptophysin-GFP (Fig. 4), supporting the idea that they behave generally similarly. However, there were a number of differences in the parameters of the models, which could thus account for the differences in the details of the results as well (Table 2).

## Discussion

### Synapse Assembly and the Phases of LTP

Collectively, these results suggest that early-phase potentiation is accompanied by a rapid increase in sites for the assembly of puncta of synaptic proteins and presynaptic structures, which may represent initial steps in the formation of new stable synapses during late-phase potentiation. The increase in sites is initiated almost immediately after the induction of potentiation, and thus can contribute to the transition between the early and late phase mechanisms of plasticity. More specifically, the new sites may serve as seeds for the formation and maintenance of new synapses, thereby acting as local “tags” for protein and RNA synthesis-dependent synaptic growth during late-phase plasticity [13], [14]. Consistent with that idea, actin polymerization, which is important for rapid changes in synaptic puncta and structures [6], [18], [21], [28], [31], [34], is also important for synaptic tagging [35]. Tagging is usually thought to contribute to the long-term strengthening of existing synapses, but our results suggest that it can contribute to *de novo* synapse formation during the late phase as well.

When the new puncta and structures become functional and whether they might also contribute to early-phase potentiation remains to be determined. Other studies have suggested that the formation of new functional synapses can take from as little as 30 min [26], [34],[36]–[39] to more than 15 hrs [28], [29], [40]. Potentiation and the increase in puncta have similar time courses and pharmacological profiles, new puncta of pre- and postsynaptic proteins colocalize, and the new presynaptic puncta undergo vesicle cycling [22], [31], suggesting that the puncta could become functional during early phase potentiation. If so, the rapid assembly of puncta of pre- and postsynaptic proteins suggests a new basis for pre- and postsynaptic “unsilencing” of synapses during early LTP [2], [41]. The rapid increase in sites where puncta may form also suggests a new type of structural mechanism (increased numbers of synapses) that has not generally been considered for early LTP.

### Synaptic Components Are Dynamic, but Sites Are More Stable

We found that the puncta and structures are very dynamic. Most of them continually assemble and disassemble on a time scale of minutes, although a subset of puncta and structures are more stable, and new puncta and structures assemble within minutes after the induction of potentiation. Previous studies of synaptic development have similarly found that some pre- and postsynaptic puncta and structures are dynamic while others are more stable, on a time scale ranging from minutes to days or weeks [7]–[12], [40], [42]–[47]. As a rule, synaptic components are more dynamic in younger preparations and become more stable in older ones. We used relatively young cultured neurons after 2–3 weeks in vitro, when there is still a fairly high level of spontaneous synapse formation. Thus our results may be relevant to activity-dependent mechanisms of synapse formation during late-stage development as well as synaptic plasticity.

Although many studies have shown that synaptic components are dynamic, it has not been known whether they assemble and disassemble at random or fixed sites, and whether plasticity alters the number or dynamics of those sites. We have found that synaptic components assemble at fixed sites that are much more stable than the components themselves. In addition, quantitative modeling suggested that rapid and long-lasting changes in the number of those sites can account for most of the changes in puncta and structures during potentiation. By contrast, our data could not be fit by changes in the rates of assembly or disassembly of the puncta or structures in the model. That result differs from several previous studies that reported a change in the rate of turnover (gains and/or losses) of synaptic components during functional plasticity [7]–[12]. However, a change in the number of sites could look like a change in the rate of turnover, and might at least partially account for those results.

Fixed sites where puncta and structures assemble and disassemble have not previously been described, perhaps because most studies follow puncta or structures over a single cycle of assembly and disassembly (although some published figures show disassembly and reassembly at the same site – e.g. ref. [11], Fig. 2A). However, the sites are similar to “pause sites” on axons where synaptic protein transport vesicles pause and where presynaptic terminals and stable contacts with postsynaptic filopodia preferentially form [48]. Some of the puncta in our study (e.g. the one indicated by an asterisk in Fig. 6A) could be transport packets of synaptic proteins [49]–[51], but two types of evidence suggest that most of our sites are not pause sites. First, we found that the appearance and disappearance of puncta of synaptic proteins is largely due to aggregation and disaggregation, rather than pausing ([21] and Figs. 1C, 5C). Second, whereas the pause sites do not require contact with a postsynaptic neuron [48], we found that puncta of pre- and postsynaptic proteins generally assemble and disassemble at shared sites (Fig. 6C). That result implies that the sites are located at points of contact between neurons, and must involve some sort of local communication between the neurons. It also implies that the sites could be functional synapses, but only when puncta of both pre- and postsynaptic proteins are present at the site simultaneously. If so, individual sites would be functional only intermittently. We are not aware of physiological evidence for intermittent transmission at individual synapses, although that result might explain slow fluctuations in the strength of synaptic connections between individual neurons [52].

### Changes in Synaptic Components during Potentiation

We observed several types of changes in presynaptic puncta and structures during long-lasting potentiation. There was a rapid (<10 min) increase in sites where puncta and structures may form, followed by a more gradual (3 hr) increase in sites. The gradual increase might be due to outgrowth of processes leading to new appositions between neurons. However, the rapid increase seems less likely to be due to outgrowth, and might instead be due to activation of dormant appositions. In addition to the increase in sites, some existing presynaptic puncta and structures were stabilized and stopped disassembling and reassembling. These two processes are reminiscent of two stages of synaptogenesis during late stage development: exuberant growth of new synapses followed by activity-dependent stabilization of some and pruning of others. Stabilization of existing puncta and structures during potentiation suggests a Hebb-type activity-dependent learning rule, in which puncta and structures that are present during the induction of potentiation (and therefore might contribute to it) are made more permanent. Recent results on postsynaptic spines support that idea [11]. However, we found that stable puncta and structures were no more likely to colocalize with other synaptic proteins, and thus are not equivalent to stable synapses.

Somewhat surprisingly, although there was a trend for a gradual increase in presumably postsynaptic structures under control conditions, we did not observe any significant changes in those structures during potentiation. That result differs from several previous studies that reported outgrowth of postsynaptic filopodia and spines following potentiation e.g. [8], [23]–[25]. However, in most of those studies the formation of new spines did not begin for 0.5–2 hrs and then had a gradual rise. Thus it is possible that under our conditions an activity-dependent increase in spines may not be apparent until after the 3 hrs that we observed them. If so, the increase in spines would lag behind the increases in presynaptic structures and both pre- and postsynaptic puncta, which all occur within 10 min. That sequence is consistent with a previous study of activity-dependent synapse formation [26], but differs from some other sequences suggested for *de novo* synapse formation e.g. [37], [48].

Alternatively, our analysis of overlapping vs. adjacent structures may not adequately discriminate between ones that are pre- or postsynaptic. For example, the overlapping structures might be predominantly postsynaptic, and adjacent structures might be nonsynaptic. However, the analysis we used has been verified by EM microscopy in other studies [33], and distinguished two clearly distinct populations of structures in this study (Fig. 9). Furthermore, the adjacent structures behaved differently than all structures (Fig. 8), many of which were presumably nonsynaptic (Fig. 7). Thus it seems more likely that the adjacent structures are postsynaptic and the overlapping structures predominantly presynaptic, as we have assumed.

### Protein Synthesis and Synapse Assembly

Protein synthesis is generally thought to be required for long-term but not more rapid structural changes during LTP and other forms of plasticity [3]. For example, whereas intermediate-term facilitation in *Aplysia* is accompanied by filling of previously empty presynaptic varicosities, which does not require protein synthesis, long-term facilitation involves stabilization of those changes as well as growth of new varicosities, both of which require protein synthesis [53]. Consistent with those results, the rapid increase in puncta during potentiation is actin dependent but not protein synthesis dependent [21], whereas the slow increase and maintenance of the rapid increase are protein synthesis dependent. Stabilization of existing puncta involves protein synthesis as well, but the direction of that effect is less clear. The dynamic behavior of puncta (but not structures) under control conditions is also protein synthesis dependent, suggesting that stabilization of the puncta might involve a *decrease* in protein synthesis.

The slow increases in both presumably presynaptic (Fig. 9 and [54], [55]) and postsynaptic [11] structures during potentiation also require protein synthesis. Unlike the puncta and mEPSCs [21], however, the rapid increase in presynaptic structures requires protein synthesis as well. Protein synthesis is similarly involved in the rapid (<10 min) as well as gradual increase in spine volume during LTP [56], and in rapid functional changes with some other LTP paradigms [57], [58] and other forms of synaptic plasticity [59], [60]. In most of those cases, the rapid changes in structure and function are thought to involve local protein synthesis. Although most vertebrate studies have focused on dendritic protein synthesis, local protein synthesis can also occur in presynaptic structures in vertebrates as well as invertebrates [61], [62].

Both local and nuclear protein synthesis are involved in induction of late-phase LTP in hippocampus [63]–[65], and play several different types of roles in the structural changes during long-term facilitation in *Aplysia* [66]–[68]. In particular, local protein synthesis is involved in “tagging” synapses for stable growth during long-term facilitation, which also requires nuclear protein synthesis [16], [66]. Local and nuclear translation may play analagous roles in the structural changes during late-phase potentiation in hippocampal neurons as well [15], [69]. If so, local protein synthesis may contribute to the rapid formation of new sites for synapse assembly, and nuclear protein synthesis may be required for their long-term maintenance.

## Materials and Methods

The experimental procedures were approved by the Institutional Animal Care and Use Committee of Columbia University. All experiments were performed on dissociated cultures of hippocampal neurons from one-day old Sprague Dawley rats. The cultures were prepared and plated on glass coverslips as described previously [70] and used 10–20 days after plating. Electrophysiological, immunocytochemical, and live imaging methods were also as described previously [21], [31], [71], [72].

Briefly, either glutamate in Mg^2+^-free bath solution or normal bath solution (control) were added directly to the culture dish and washed out after approximately 1 min. Anisomycin was applied for 1 hr before and during the glutamate application and washed out with the glutamate. Puncta of synaptophysin-IR, GluR1-IR, synapsin-IR, and synaptophysin-GFP were examined with a MRC-1000 laser confocal scanning system coupled to a Zeiss Axiovert inverted microscope and analyzed automatically using a computer program written in IDL (Research Systems, Inc.). The puncta were identified based on having a fluorescence intensity that exceeded a threshold set by a blind observer to maximize discrimination of puncta from the background, and a diameter between 0.5 and 5 µm. In live imaging experiments, once the fluorescence intensity threshold, laser intensity, and photo-multiplier gain were set, they were not changed for the remainder of the experiment. Small structures on processes of GFP expressing neurons were identified by subtracting a smoothed image from the original image and then analyzed similarly to puncta of synaptophysin-GFP, except that the size range was between 0.5 and 3 µm. Structures and puncta of different types were said to be colocalized if their boundaries overlapped or came within 1 µm of each other, and were said to be adjacent if they colocalized but did not overlap.

A representative field was imaged before, 10 min, 1 hr, and 3 hrs after the glutamate application and the number of puncta or structures at each time was normalized to the number before glutamate application (pretest) in each experiment. In addition, the “life histories” of the puncta or structures were analyzed by superimposing the images from the four times and counting puncta or structures that colocalized at each possible combination of times (history). We did not analyze changes in size. In some experiments, the neurons were fixed after the last image and processed for GluR1 or synapsin-I immunocytochemistry. A computer controlled motorized microscope stage was then used to take another image of the same field, and the subset of puncta or structures that colocalized with GluR1-IR or synapsin-IR puncta were analyzed as described for all puncta or structures. The data were analyzed with 3-way ANOVAs with one repeated measure (time or history) followed by planned comparisons of the different experimental treatments at each time or history [73]. If the direction of the effect was predicted, one-tail tests were used. P<0.05 is considered significant.

Cells expressing mouse PSD-95 fused to RFP (Red Fluorescent Protein: dsRed2, Clontech) were excited using the 568 nm line of a krypton-argon laser. The field was imaged before, 10 min, and 30 min after saline application (control), and changes in puncta were analyzed similarly to puncta of synaptophysin-GFP. In some experiments, changes in fluorescence intensity in the area bounded by the puncta and the surrounding area within 5 µm were analyzed with paired t-tests. In experiments with coexpression of synaptophysin-GFP and PSD95-RFP their joint life histories were analyzed by superimposing images of the two types of puncta at two times, and counting puncta that colocalized in each possible combination of the four images.

In modeling experiments, the parameters were chosen to minimize the summed absolute deviations of the model from the mean data on the time courses and life histories of the puncta or structures in each experimental condition. This process was repeated during several iterations of an exhaustive search of the parameter space at increasingly higher levels of resolution. The model was said to fit the actual data very well if the results of the model were within 1 SEM of the data in most of the experimental conditions (35 out of 36 in both Figs. 4B,C and 10B,C), and not significantly different from the data in any condition. Parameters in the model were said to be similar if they were within a range that produced little difference in the overall fit (i.e., within about 10 for N1 and N2, 0.10 for P1 and P2, and 0.010 for P3).
